# Functional and transcriptomic characterization of carboplatin-resistant A2780 ovarian cancer cell line

**DOI:** 10.1186/s40659-019-0220-0

**Published:** 2019-03-21

**Authors:** Tamara Viscarra, Kurt Buchegger, Ignacio Jofre, Ismael Riquelme, Louise Zanella, Michel Abanto, Alyssa C. Parker, Stephen R. Piccolo, Juan Carlos Roa, Carmen Ili, Priscilla Brebi

**Affiliations:** 1 Laboratorio de Patología Molecular, Centro de Excelencia en Medicina Traslacional-Scientific and Technological Bioresource Nucleus (CEMT-BIOREN), Universidad de La Frontera, Avenida Alemania #0478, 3th Floor, Temuco, Chile; 2 Laboratory of Neurosciences and Biological Peptides, Center of Biotechnology in Reproduction (CEBIOR-BIOREN), Department of Preclinical Sciences, Faculty of Medicine, Universidad de La Frontera, Temuco, Chile; 3 Instituto de Ciencias Biomédicas, Facultad de Ciencias de la Salud, Universidad Autónoma de Chile, Temuco, Chile; 4 Scientific and Technological Bioresource Nucleus (BIOREN), Universidad de La Frontera, Casilla 54-D, Temuco, Chile; 5 Department of Biology, Brigham Young University, Provo, UT USA; 6 Department of Pathology, UC Centre for Investigational Oncology (CITO), Advanced Centre for Chronic Diseases (ACCDis), The Millennium Institute on Immunology and Immunotherapy, Pontificia Universidad Católica de Chile, Santiago de Chile, Chile

**Keywords:** A2780 cell line, Carboplatin, Drug resistance, Wnt/β-catenin-signaling pathway, Integrin signaling pathway, Ovarian cancer

## Abstract

**Background:**

Ovarian cancer is a significant cancer-related cause of death in women worldwide. The most used chemotherapeutic regimen is based on carboplatin (CBDCA). However, CBDCA resistance is the main obstacle to a better prognosis. An in vitro drug-resistant cell model would help in the understanding of molecular mechanisms underlying this drug-resistance phenomenon. The aim of this study was to characterize cellular and molecular changes of induced CBDCA-resistant ovarian cancer cell line A2780.

**Methods:**

The cell selection strategy used in this study was a *dose*-*per*-*pulse* method using a concentration of 100 μM for 2 h. Once 20 cycles of exposure to the drug were completed, the cell cultures showed a resistant phenotype. Then, the ovarian cancer cell line A2780 was grown with 100 μM of CBDCA (CBDCA-resistant cells) or without CBDCA (parental cells). After, a drug sensitivity assay, morphological analyses, cell death assays and a RNA-seq analysis were performed in CBDCA-resistant A2780 cells.

**Results:**

Microscopy on both parental and CBDCA-resistant A2780 cells showed similar characteristics in morphology and F-actin distribution within cells. In cell-death assays, parental A2780 cells showed a significant increase in phosphatidylserine translocation and caspase-3/7 cleavage compared to CBDCA-resistant A2780 cells (P < 0.05 and P < 0.005, respectively). Cell viability in parental A2780 cells was significantly decreased compared to CBDCA-resistant A2780 cells (P < 0.0005). The RNA-seq analysis showed 156 differentially expressed genes (DEGs) associated mainly to molecular functions.

**Conclusion:**

CBDCA-resistant A2780 ovarian cancer cells is a reliable model of CBDCA resistance that shows several DEGs involved in molecular functions such as transmembrane activity, protein binding to cell surface receptor and catalytic activity. Also, we found that the Wnt/β-catenin and integrin signaling pathway are the main metabolic pathway dysregulated in CBDCA-resistant A2780 cells.

**Electronic supplementary material:**

The online version of this article (10.1186/s40659-019-0220-0) contains supplementary material, which is available to authorized users.

## Background

Ovarian cancer is the sixth most common cancer and the seventh highest cause of cancer death in women worldwide. However, the incidence of this cancer varies by geographical region, with Europe and North America having the highest incidence rates, while the lowest incidence has been observed in China and African nations [1]. Epithelial ovarian cancer (EOC) comprises about 90% of all ovarian tumors, whereas stromal tumors and germ cell tumors comprise the remaining 10% [2]. The standard treatment consists in the partial removal of the tumor followed by a chemotherapeutic scheme based on platinum drugs and taxanes [3]. Some clinical trials of advanced ovarian cancer have documented that carboplatin has equivalent antitumoral activity to cisplatin, but with considerably less side effects [4]. In this regard, the randomized evidence on the efficacy and toxicity associated with the regimens used in several trials concludes that the use of carboplatin (CBDCA)-as a platinum drug—is a safe and effective first-line treatment for women with advanced ovarian cancer [5–7]. Despite the effective response with platinum drugs and taxanes, the 5-year survival rate in EOC patients is around 20–30% in advanced stages (II–IV) [8]. This low survival rate has been largely related to the resistance of certain ovarian cancer cells to a wide range of chemotherapeutic drugs, being considered the main obstacle to a better prognosis [9]. In this context, several pathways have been identified as possibly responsible for the resistant phenotype, including drug uptake, recognition of DNA damage, DNA repair and apoptosis process. However, no metabolic pathway has been reliably associated with CBDCA-resistance in ovarian cancer [10].

For this reason, the establishment of an in vitro cellular model of resistant ovarian cancer is needed to respond to several questions related to platinum drug resistance. In this regard, previous reports have established two major methodological approaches for developing drug-resistant cell lines in vitro: (1) the clinically relevant models and (2) the high-level laboratory models. Depending on the chosen method, the development of a drug-resistant cell line can take from 3 to 18 months [11]. The clinically relevant models try to mimic the conditions that cancer patients experience during chemotherapy: a pulsed treatment strategy with lower drug doses is often used along with a short cell recovery time with drug-free medium.

Yet despite knowing the methodology used to establish these models, there is scarce knowledge about the morphological, cellular and molecular characteristics of an in vitro resistant phenotype. The evaluation of several parameters in induced drug-resistant cells lines could facilitate the differentiation between a real drug-resistant cell from a stressed cell, making possible to obtain a reliable and reproducible model for drug resistance research. The aim of this study was to characterize the CBDCA-resistant ovarian cancer cell line A2780 functionally and molecularly through a clinically relevant methodology (*dose*-*per*-*pulse* method).

## Results

### Sensitivity to carboplatin in parental and CBDCA-resistant A2780 cells

**Fig. 1:**
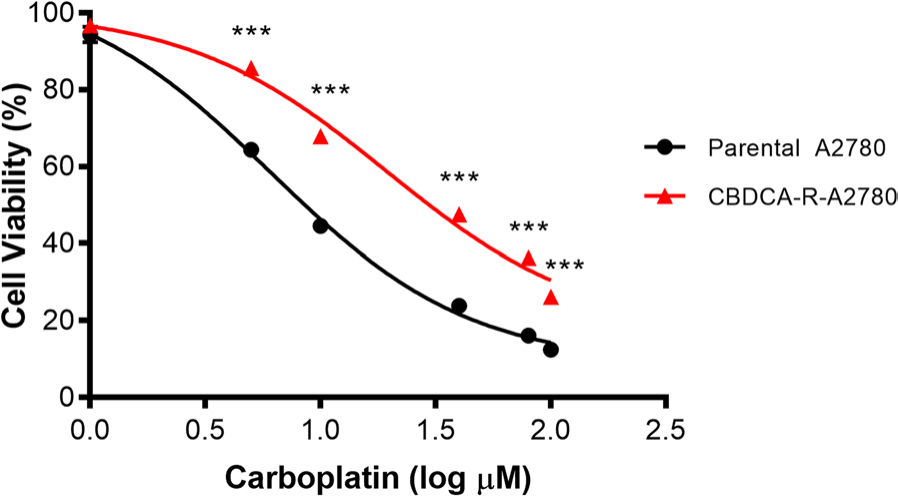
The EC_50_ values for cell viability in parental A2780 cells from CBDCA-resistant A2780 cells. EC_50_ values were calculated using mathematic function antilog of values provided by sigmoidal dose–response curves. Antilog _EC50 A2780-parental_ (0.78 log µM) = 6.05 µM; Antilog _EC50 A2780-CBDCA_ (1.29 log µM) = 19.35 µM. ***P < 0.001

The establishment of a carboplatin resistance model in an A2780 cell line (CBDCA-resistant A2780) was obtained after 16 months of exposure to doses per pulse of CBDCA (specified in Methods section). After 2 months of freezing, sensitivity to CBDCA was examined by comparing parental A2780 cells from CBDCA-resistant A2780 cells. For this purpose, we evaluated the effective concentration that causes 50% cell death (EC_50_). The EC_50_ for the parental A2780 cells was obtained at concentration of 6.05 μM ± 1.08 (0.78 ± 0.035 log μM) of CBDCA while the EC_50_ for CBDCA-resistant A2780 cells was established at a concentration of 19.35 μM ± 1.16 (1.29 ± 0.065 log μM) of CBDCA (Fig. 1). The resistance index for CBDCA-resistant A2780 cells was 3.2-fold higher than parental A2780 cells.

### Morphological comparisons between parental and CBDCA-resistant A2780 cells

**Fig. 2:**
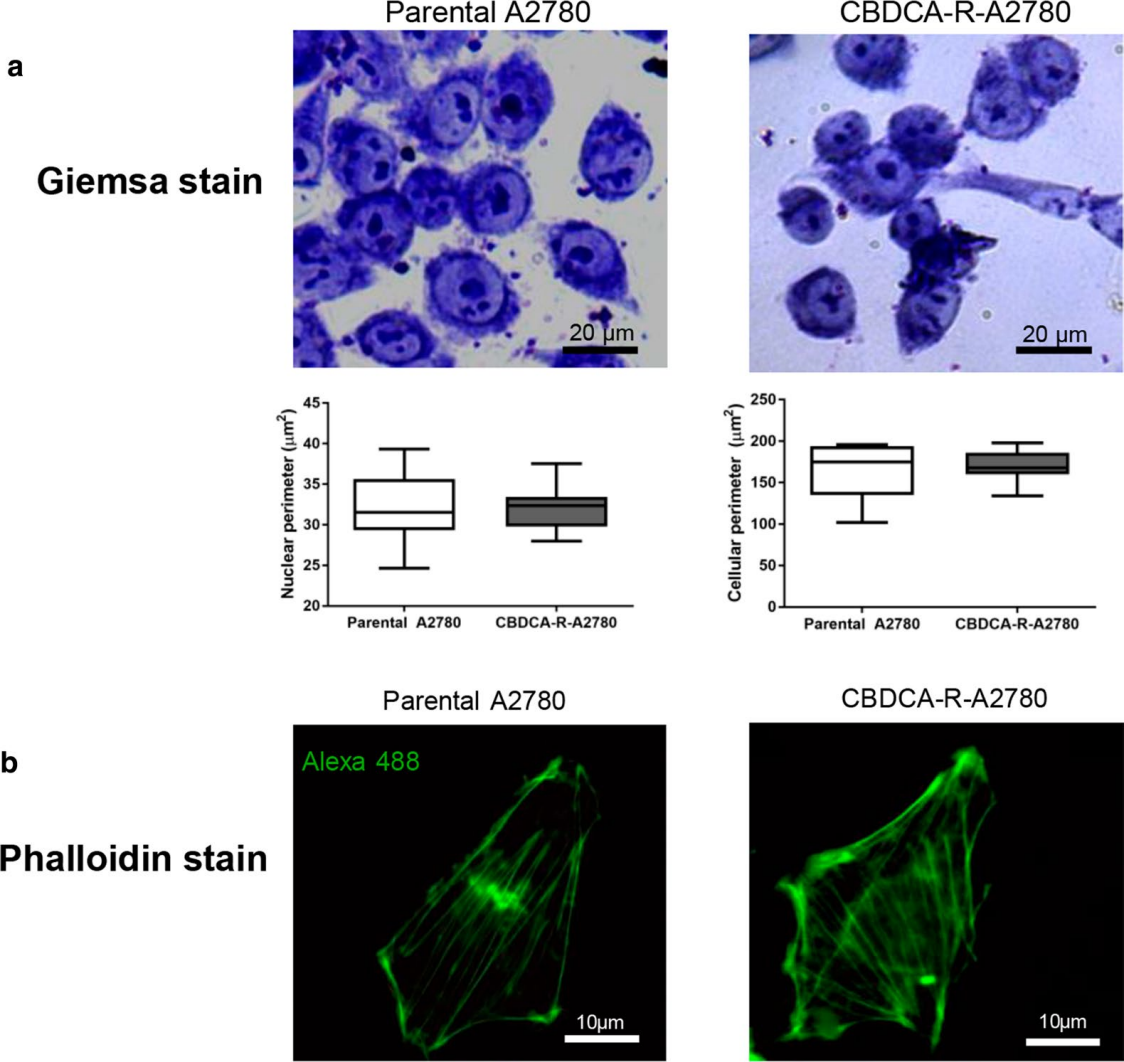
Morphological comparisons between parental and CBDCA-resistant A2780 cells. **a** Giemsa staining and ImageJ analysis for morphometric observation according to the cellular and nuclear perimeter of each cell line. **b** Distribution of F-actin in both conditions. No significant differences were observed according to morphological features between parental and CBDCA-resistant A2780 cells

We evaluated cell morphology in both conditions. Giemsa staining and ImageJ analysis showed no significant differences according to cell perimeter and nuclear perimeter in either parental or CBDCA-resistant A2780 cells (Fig. 2a). Likewise, F-actin distribution within cells was the similar in both conditions (Fig. 2b).

### Response to CBDCA-induced cell death in both parental and CBDCA-resistant A2780 cells

**Fig. 3:**
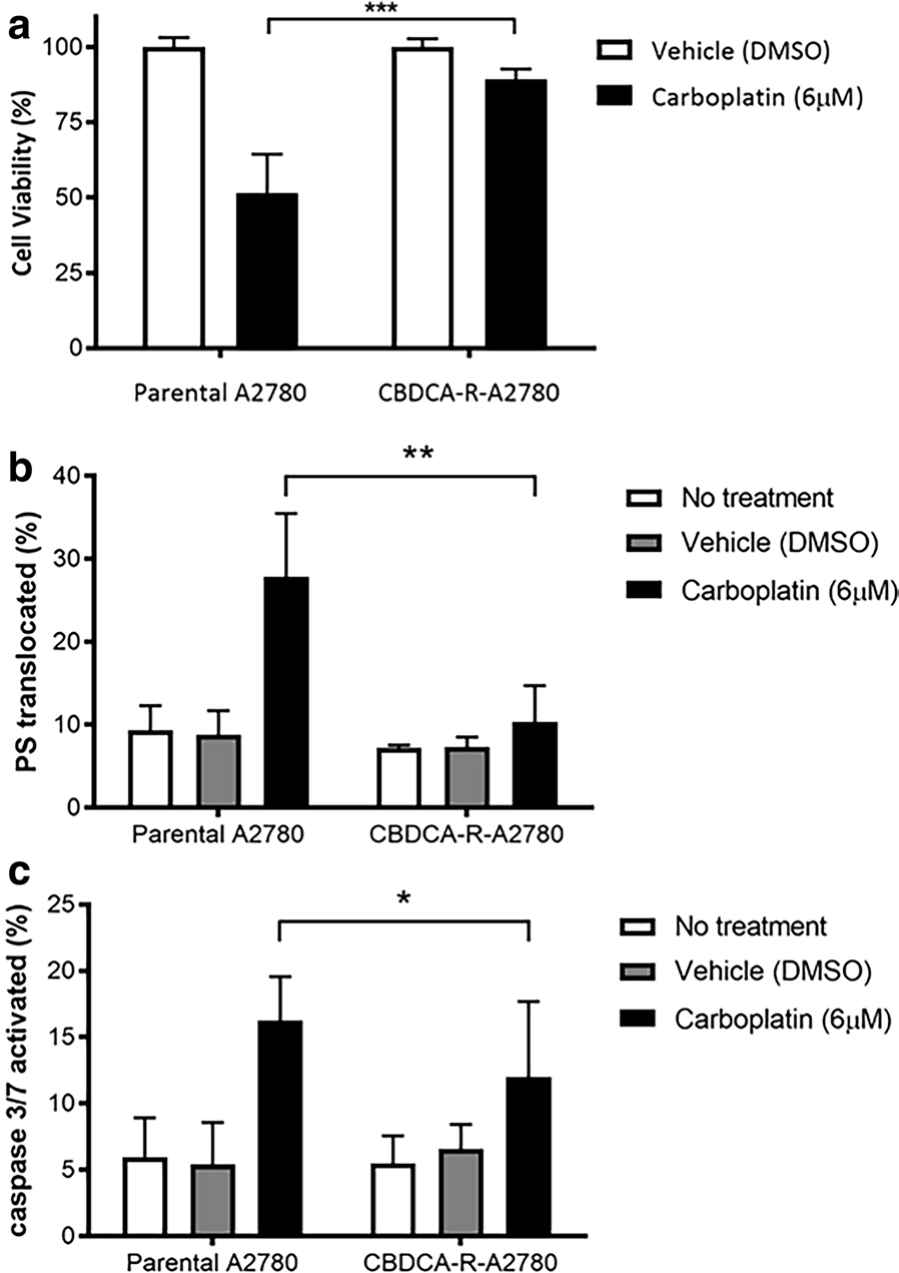
Effect of CBDCA exposure in the viability and cell death of parental and CBDCA-resistant A2780 cells. **a** Cell viability. **b** Phosphatidylserine (PS) translocation. **c** Caspase-3/7 cleavage. These results confirm the CBDCA resistant phenotype of CBDCA-resistant A2780 cells. *P < 0.05; **P < 0.005; ***P < 0.0005

After establishing the concentration of drug necessary to generate 50% cell death in parental and CBDCA-resistant A2780 cells, we used a concentration of 6.05 μM ± 0.123 μM for 72 h for subsequent tests in both conditions. The cell viability assay showed that CBDCA exposure significantly decreased cell viability in parental A2780 cells compared to the CBDCA-resistant A2780 cells (P < 0.001) (Fig. 3a).

Next, we examined the differences in the cell death effect induced by CBDCA treatment between parental and CBDCA-resistant A2780 cells, thereby phosphatidylserine (PS) translocation and caspase-3/7 cleavage assays were performed. After exposure with CBDCA, the parental A2780 cells showed a significant increase in PS translocation (mean = 29.26% ± 7.6%) compared to CBDCA-resistant A2780 cells (mean = 13.16% ± 4.4%) (Fig. 3b, P < 0.005). Similarly, parental A2780 cells showed a significant increment in the cleavage of caspases 3/7 (mean = 17.46% ± 3.3%) compared to CBDCA-resistant A2780 cells (mean = 10.48% ± 2.8%) (Fig. 3c, P < 0.05). In addition, within the CBDCA-resistant A2780 cells no significant differences in these parameters were found in untreated vehicle (DMSO) and CBDCA (6 μM) conditions. As expected, these results confirm that CBDCA-resistant A2780 cells effectively acquired a drug-resistant phenotype compared to parental A2780 cells.

### Transcriptomic sequencing analysis in parental A2780 and CBDCA-resistant A2780 cells

**Fig. 4:**
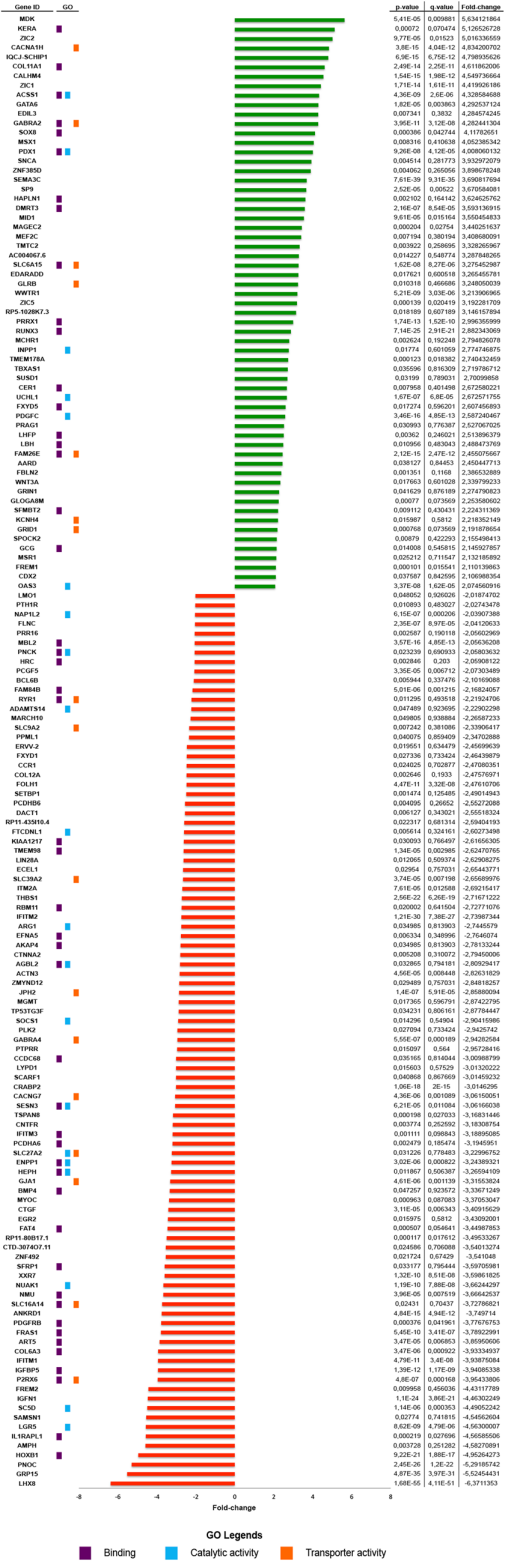
Differentially expressed genes (DEGs) in carboplatin-resistant A2780. Upregulated (green bars) and downregulated (red bars) genes are ordered according to fold-change value. Gene ontology is shown according to the three main categories: *binding* (purple dots), *catalytic activity* (light blue dots) and *transporter activity* (orange dots). Table shows P-value, q-value and fold-change for each gene

In order to identify differentially expressed genes (DEGs) that are relevant to the chemoresistant phenotype in ovarian cancer cell lines, we performed an expression analysis on parental A2780 and CBDCA-R-A2780 cell lines using RNA-seq. A total of 14.673 protein-coding genes were sequenced. From those, 156 transcripts showed deregulated expression; 95 were downregulated and 61 were upregulated in CBDCA-R-A2780 compared to A2780-sensitive (Fig. 4; Additional file 1: Table S1).

### Gene ontology analysis

Gene ontology (GO) analysis from those DEGs were performed classifying the mRNAs according to molecular function.

**Fig. 5:**
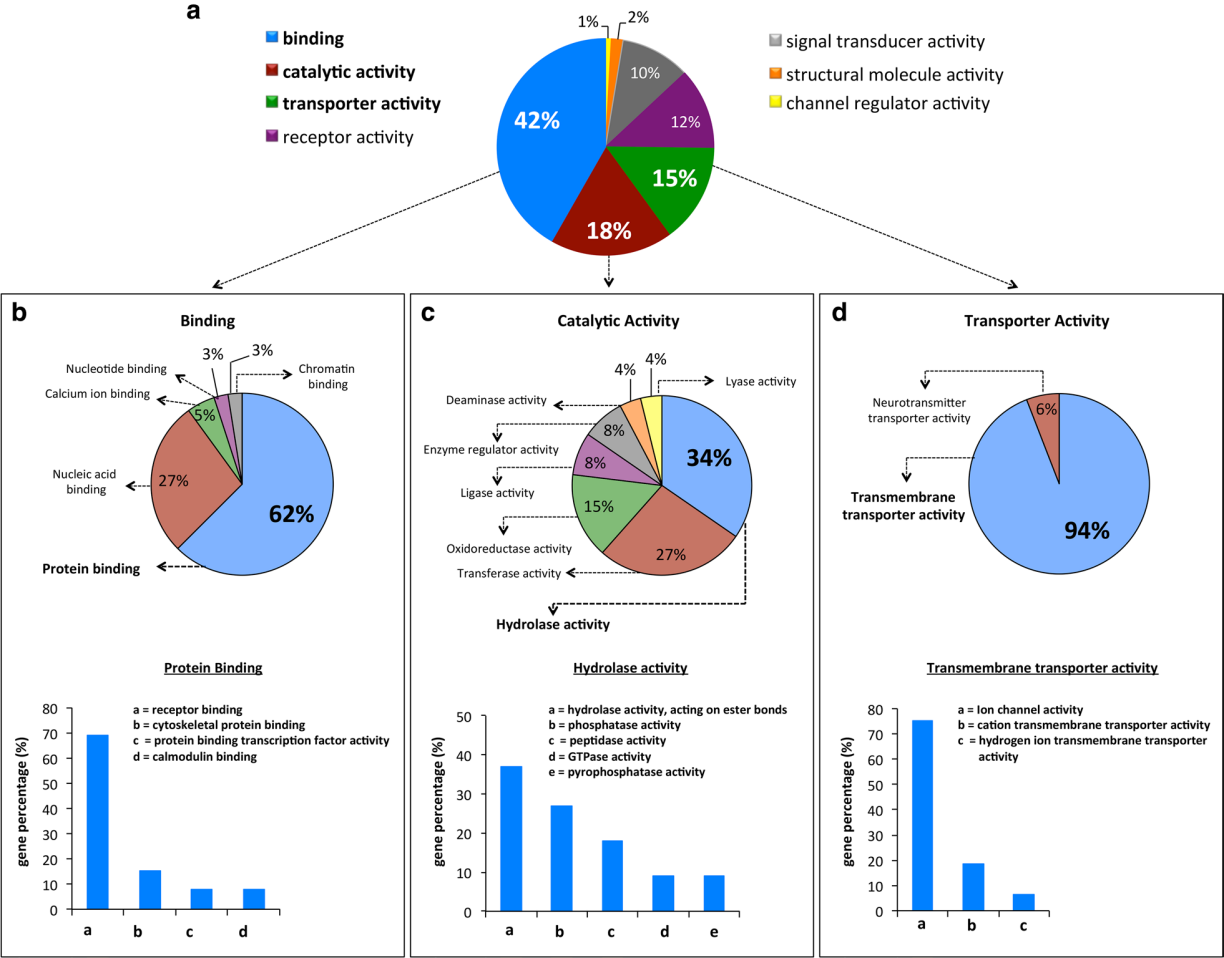
Gene ontology (GO) analysis of the DEGs. Pie charts represent the enriched GO: term associated to DEGs. **a** GO categories. **b** Binding category and its respective ontological subcategories. **c** Catalytic activity category and its respective ontological subcategories. **d** Transporter activity category and its respective ontological subcategories

Regarding the molecular function category, the transcripts were mainly associated to *binding activity* (42%), *catalytic activity* (18%) and *transporter activity* (15%) categories (Fig. 5a). Within the *binding activity* category (mRNAs that encode for proteins that bind other molecules such as proteins, DNA, RNA, etc.), many transcripts were found to be associated with *protein binding* (62%), and within this GO subcategory, the main ontology was found for *receptor binding* function (69%) (Fig. 5b). The GO category of *catalytic activity* showed 34% of transcripts involved in *hydrolase activity* mainly associated to *hydrolase activity, acting on ester bonds* (37%), *phosphatase activity* (27%) and *peptidase activity* (18%) subcategories (Fig. 5c).

Within *transporter activity* category, we found an enrichment of transcripts associated with *transmembrane transporter activity* (94%), which are mainly represented by transcripts that encode transmembrane proteins with *ion channel activity* (75%) (Fig. 5d).

### Molecular pathway analysis

**Fig. 6:**
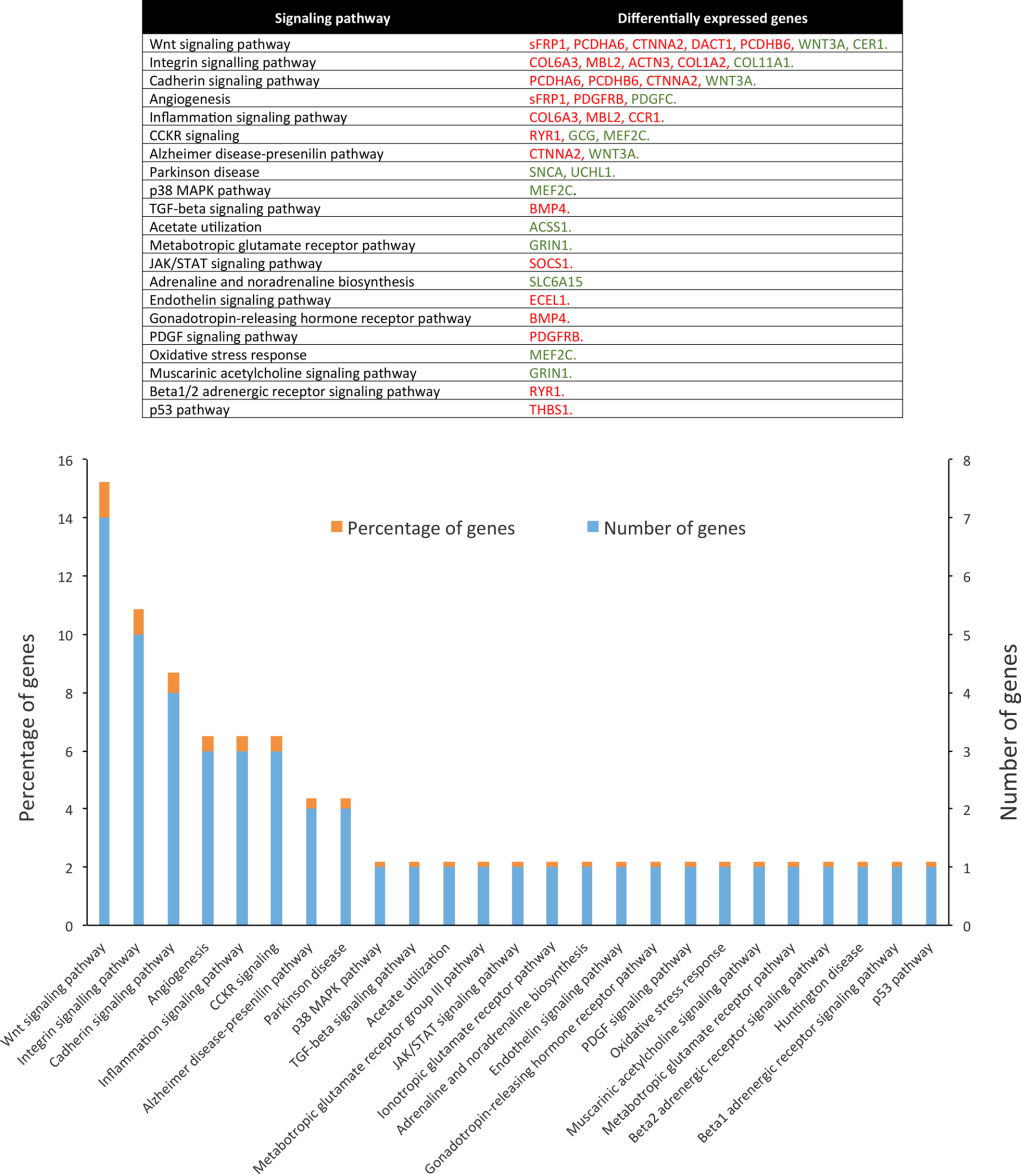
Molecular pathways analysis of DEGs. The table shows the signaling pathways deregulated in CBDCA-R-A2780 and their associated DEGs. In the graph, the vertical axes show the percentage of genes (orange) and number of genes (blue) annotated for each signaling pathway. Horizontal axis shows the name of the 21 deregulated metabolic pathways

In the present study, from 156 DEGs only 149 were classified into 21 metabolic pathways. Among these pathways, the most enriched metabolic pathways were Wnt/β-catenin and integrin signaling pathway. The Wnt/β-catenin signaling pathway was the most enriched with 15.2% of DEGs, which contains 5 downregulated DEGs (*sFRP1, PCDHA6, CTNNA2, DACT1*, and *PCDHB6* genes) and 2 upregulated DEGs (*WNT3A* and *CER1* genes). Meanwhile, integrin signaling pathway was the second most enriched with 10.9% of DEGs, which are 4 downregulated DEGs (COL6A3, MBL2, ACTN3, COL1A2) and 1 upregulated DEGs (COL11A1) (Fig. 6).

## Discussion

The most common chemotherapeutic treatment for ovarian cancer is based on the use of platinum drugs—specifically carboplatin—in combination with paclitaxel. This combination is the most common treatment for high-grade serous ovarian cancer [12]. Although chemotherapy is the preferred treatment modality, the development of chemoresistance is the main problem that limits treatment success and patient’s prognosis [13]. The mechanisms involved in platinum drug resistance are partially known and probably multifactorial in origin [14]. Therefore, the need to study these mechanisms has become pivotal to understanding the chemoresistant phenotype. In this article, a reliable ovarian cancer model is offered to assess many of these features of interest, including transcriptomic, gene ontology and signaling pathway analyses.

As expected, the CBDCA-resistant cells clearly showed 3.2-fold of increase in the CBDCA resistance index compared to parental A2780 cells. Although this value can be considered a reliable drug resistance marker, the fold-resistance value should not be compared to other in vitro chemoresistance models because each cell line is biologically different and each resistance model has been developed using diverse drug administration methodologies [11, 15].

In previous works conducted by Li et al. and Yan et al. [11, 16] developed a carboplatin-resistant A2780 cell line. However, the treatment established by them is considered a “high level laboratory model” described by McDermott et al. [11]. In which the development of the model is based on the addition of drugs in a continuous way increasing its concentration in each cycle. The gradual increases of these concentrations exceed the limit allowed in clinical due to its high cytotoxicity [11, 17].

On the other hand, in this work we developed the “clinically relevant method”, which seeks to emulate the treatment conditions of patients during chemotherapy where a cellular recovery time (drug-free) is usually required, emulating the resting periods of the patient (treatment-free). The main advantages and disadvantages are that the high-level laboratory model achieve higher resistance index levels than the clinically relevant method. However, the main disadvantage of these models is the higher the level of resistance, the less relevant the model becomes to the clinic [11].

On the other hand, parental and CBDCA-resistant A2780 cells in culture showed similarities in morphology, which suggests that CBDCA-resistant cells do not undergo major structural changes once the drug-resistant phenotype has been acquired. In this regard, some reports associated to platinum-drug resistance in ovarian cancer cells have shown no apparent morphological differences in each condition [18–20], whereas in other models, cell morphology is gradually recovered—similar to the parental condition—in the logarithmic phase [8]. This is probably because cells differ in their morphology due to cell stress caused by the drug.

In addition, cell viability assays and cell death analyses demonstrated that CBDCA-resistant A2780 cells effectively acquired an advantage in survival features after drug exposure compared to parental cells. These higher survival rates are likely attributable to inhibition or evasion of apoptotic processes developed when CBDCA resistance is reached [21, 22]. In this sense, several factors may be considered within these alterations to apoptotic processes in CBDCA-resistant A2780 cells, being the off-target resistance mechanism the most commonly implicated, and it is characterized by alterations in signaling pathways that interfere with pro-apoptotic events induced by platinum drugs [23, 24]. For example, the activation of canonical Wnt signaling has been involved in the inhibition of the cytochrome C release and the subsequent caspase-9 activation induced by chemotherapeutic drugs [25–27].

The transcriptome analysis of our isogenic model for CBDCA resistance showed 156 DEGs involved in subcategories of molecular functions, such as *protein binding* to cell surface receptors, *hydrolase activity* and *transmembrane transporter activity*. In this regard, some of the ontology classifications showed in this report have a relation with the drug-resistant phenotype. In this study, the most interesting DEGs are those associated with *transmembrane transporter activity* subcategory. Interestingly, transmembrane transporters govern the movement of drugs and their metabolites across biological membranes, thereby determining the pharmacokinetics, efficacy and adverse drug reactions [28]. Ion channels are integral membrane proteins that allow the passive diffusion of certain ions into and out of the cell maintaining intracellular ionic homeostasis needed to all basic cellular processes and also in the malignant phenotype of cancer cells [29, 30]. In cancer drug resistance, some of the most studied mechanisms are drug influx and efflux through transmembrane channels [31]. These channels decrease the intracellular drug accumulation as result of a decrease in drug influx via drug solute carriers or an increase in drug efflux via ATP-binding cassette (ABC) pumps [32–34]. In platinum-drug resistance reduced accumulation of Platinum compounds in the cytosol is caused by reduced uptake, increased efflux, or cellular compartmentation. Several ABC transport proteins are involved, including MRP2 and MRP6, Ctr1 and Ctr2, ATP7A and ATP7B [35–37].

Additionally, chemoresistance can be triggered by overexpression of receptor tyrosine kinases: ERBB1-4, IGF-1R, VEGFR 1-3, Wnt receptor and PDGF receptor family members [38, 39].

Another mechanism involved in chemoresistance is drug inactivation. In this regard, GO analysis demonstrated that many DEGs also have *catalytic activity*, which are enriched by several specific functions such as: hydrolase, transferase, oxidoreductase, among others. The hydrolysis reaction of carboplatin is necessary to activate this molecule once it enters into the cell [40]. In the cytoplasm, platinum agents becomes aquated, which then enables them to react with thiol-containing molecules, including glutathione and metallothioneins [41]. Increased glutathione levels may cause resistance by binding/inactivating platinum drug, enhancing DNA repair, or reducing cisplatin-induced oxidative stress [42]. Cisplatin is detoxified by glutathione through adduct formation [43]. An additional member of the antioxidant defense system is thioredoxin, which regulates the oxidation reduction environment of the cell in a similar manner to glutathione [41, 44]. However, the information available about carboplatin detoxification is scarce and in most studies extrapolates the observations made over the cisplatin metabolization process.

In this study, in silico analyses showed that the most deregulated metabolic pathways in CBDCA-R-A2780 were Wnt/β-catenin and integrin signaling pathway. Canonical activation of Wnt signaling—mediated through β-catenin—has been shown to be a critical regulator of chemoresistance in many tumors [45–51]. For instance, an integrative study using The Cancer Genome Atlas (TCGA) data identified the upregulation of Wnt/β-catenin signaling pathway in ovarian tumors with poor prognosis [52]. In addition, Nagaraj et al. [39] studied the role of Wnt/β-catenin signaling in the regulation of cisplatin resistance and stem-like properties using cisplatin-resistant A2780 (A2780cis) cells and a primary cell culture obtained from ascites of a patient with high-grade serous ovarian cancer. They found that Wnt/β-catenin signaling pathway acts as a novel driver of cisplatin resistance by maintaining stem-like properties in ovarian cancer [39].

On the other hand, negative regulators of the Wnt/β-catenin signaling cascade have also been shown to be important for acquiring cisplatin sensitivity. For example, the addition of recombinant protein secreted frizzled-related protein 4 (sFRP4) has been positively associated with response to cisplatin and doxorubicin in cancer stem cells (CD133^+^/CD44^+^) isolated from A2780 ovarian cancer cell line [53]. This is likely because the presence of sFRP4 has been demonstrated to confer chemo-sensitization and improve chemotherapeutic efficacy through the inhibition of Wnt/β-catenin signaling [54]. Also, the overexpression of DACT1 -an antagonist of Wnt/β-catenin pathway—has been able to inhibit tumor growth and cisplatin resistance in type I epithelial ovarian cancer [55]. Likewise, low molecular weight heparin had the capability to re-sensitize the A2780cis human ovarian cancer cells through an inhibitory effect over Wnt-signaling pathway [56].

In a study conducted by Barghout et al. in A2780cis cells they found that elevated β-catenin activity contributes to carboplatin resistance in an in vitro model of human ovarian cancer [57]. These results support our finding about deregulation of Wnt/β-catenin in ovarian cancer resistant to carboplatin. However, this cell lines were resistant to cisplatin with cross-resistance to carboplatin. Unfortunately, they did not perform a high throughput analysis in order to identify others metabolic pathway deregulated. In this regard, our study identified at transcriptional level, the integrin signaling pathway deregulated. In this case, upregulation of Collagen Type 11 Alpha 1 (COL11A1) was interesting. In normal conditions expression of collagen 11 is very low or nonexistent in most tissues [58]. However, is overexpressed at mRNA and protein levels in many cancer types [59]. In ovarian cancer its overexpression has been associated to poor prognosis, metastasis and drug resistance [60–62]. COL11A1 has been reported as a cisplatin resistant marker for EOC in several recent studies [63, 64]. In A2780cis cells, COL11A1 is an important determinant of chemoresistance to cisplatin and paclitaxel through activation of Akt/c/EBP β and stabilization of PDK1 protein preventing its ubiquitination and proteasomal degradation protecting the cells from drugs cytotoxic effect [65]. Another study show that COL11A1 can upregulate IKKb transcription to constitutively activate the NF-kB-signaling pathway, thereby promoting TWIST1 and Mcl-1 expression, which were associated with chemoresistance and apoptosis inhibition [66].

In summary, the targeted inhibition of Wnt/β-catenin signaling and COL11A1 (an upregulated gene within integrin signaling pathway) seems to increase the sensitivity to platinum and taxane agents in cancer by deregulating not only Wnt/β-catenin pathway (per se) but also AKT and NF-kB pathways. Therefore, the inhibition of these metabolic pathways appears to be a promising therapeutic alternative in ovarian cancer and other malignancies resistant to platinum drugs, including cisplatin and carboplatin [39, 65–68].

## Conclusion

In summary, our study is the first report about the development and phenotypical and molecular characterization of a carboplatin-resistant ovarian cancer cell line, in order to achieve a reliable model to be used in the study of carboplatin resistance in this malignancy. In addition, several DEGs have been found to be involved in drug influx/efflux and drug hydrolysis processes and could be suggested as potential molecular markers associated with carboplatin resistance. Finally, bioinformatics analyses have been shown that Wnt/β-catenin and integrin signaling pathway may have a direct effect on the platinum resistance, by itself or an effect as a whole, so that the therapeutic success may be benefited by the joint inhibition of these metabolic pathways and constituting as a potential therapeutic alternative in cases of ovarian cancers resistant to carboplatin with Wnt/β-catenin activated and/or COL11A1 overexpressed. However, these findings are necessary to validate in future studies both in vitro and in vivo.

## Methods

### Cell culture

Ovarian cancer cell line A2780 (derived from ovarian epithelial carcinoma of an untreated patient) was kindly provided by Dr. Gareth Owen, Department of Physiology, Pontificia Universidad Católica de Chile, Santiago. Cells were grown in RPMI 1640 medium (ATCC modification) (Gibco, Thermo Fisher, USA) supplemented with 10% fetal bovine serum (FBS) (Hyclone, Thermo Fisher, USA), 2 mM glutamine (Corning, USA) and 1% penicillin/streptomycin (Corning, USA) and maintained at 37 °C in a humidified atmosphere at 5% CO_2_.

### Chemicals

Carboplatin (CBDCA) was purchased from Selleckchem, USA and suspended and stocked at a concentration of 40 mM in DMSO and then dispensed in microcentrifuge tubes with 20 µL of total volume per tube. The working stock was stored at − 20 °C for a maximum period of 1 month, while the stock tubes were maintained at − 80 °C. The working solution for each test was prepared at the time of use at a final concentration of 100 μM diluted in culture medium.

### Cell selection strategy outline

A2780 cells were seeded in a 6-well plate at a cell density of 2 × 10^5^ cells per well. Once the cell culture had a confluence of 70%, complete medium including 100 μM of CBDCA was added to the respective wells and incubated for 2 h at 37 °C with 5% CO_2_. Subsequently, culture medium was replaced with fresh drug-free culture medium. Once the cell culture reached a 90% confluence (recovery period), the cells were subcultured in drug-free medium and cultured until reaching a confluence of 70% again for the next exposure to the drug.

**Fig. 7:**
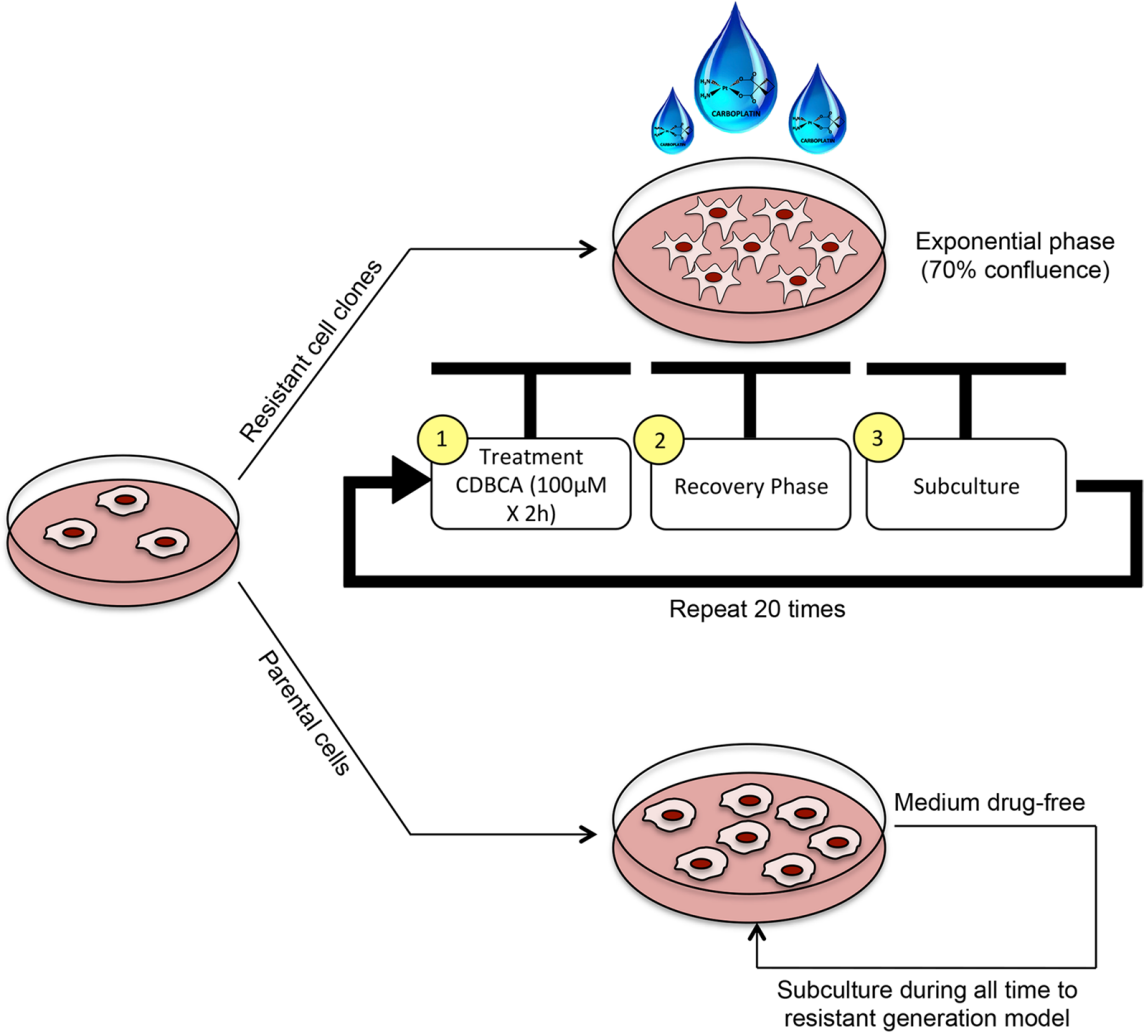
Workflow used in the development of CBDCA-resistant A2780 cells

In summary, each cycle of drug exposure consisted of two steps: (1) A2780 cell incubation with CBDCA (100 μM for 2 h) in the exponential growth phase and (2) drug removal and replacement by fresh drug-free culture medium to allow the recovery of cell confluence. These cycles were repeated until a total number of 20 cycles was reached (Fig. 7). Then, cells were frozen for 2 months, thawed for cytotoxicity analysis and compared to parental cells in order to calculate fold-resistance.

### Drug sensitivity assay

A sensitivity assay and post-determination of effective concentration to reduce the 50% of population (EC_50_) value in parental A2780 cells (sensitive to CBDCA) and CBDCA-resistant A2780 cells were determined using the MTT-formazan assay (Sigma Aldrich, USA). A number of 1 × 10^4^ cells were seeded in a 96-well plate using complete media with 10% FBS and allowed to attach overnight. After cell attachment, increasing concentrations of CBDCA were added to appropriate wells. Cells were incubated for 24, 48 and 72 h after the CBDCA treatment. Afterwards, 20 μL of MTT solution was added into each well, according to the manufacturer’s protocol. Then, cells were incubated for 2 h at 37 °C. Finally, one volume (100 μL) of isopropanol was added to diluted the formazan crystals. Absorbance was measured in a spectrophotometer at 570 nm. The viability analysis was normalized using the maximum and minimum data observed in measures.

### Morphological observations

Giemsa staining was used to determine differences between parental A2780 and CBDCA-resistant A2780 cells in both cellular and structure morphology. The cells were cultured on gelatinized coverslips (gelatin 2%) for 24 h at 37 °C. The, the cells were stained using 2% Giemsa stain (Sigma Aldrich, USA), washed twice with PBS and mounted on slides by adding mounting solution (Fluorescence Mounting Medium, DAKO, USA). Slides were observed and photographed using a camera mounted to an optical microscope. Morphological parameters such as the cellular and nuclear perimeter were analyzed using Image J (v. 1.39) with MacBiophotonics plugins.

Otherwise, the distribution of F-actin filaments was evaluated by labeling this molecule with Alexa 488-Phalloidin (Life technologies, USA). Next, the cells were carried out on gelatinized coverslips, as described previously. Then, the specimens were fixed in formaldehyde (3.7%) for 10 min at room temperature and washed with 1× PBS. Cell permeabilization was performed using 0.1% Triton X-100 and incubated for 5 min at − 20 °C. Subsequently, samples were labeled with Phalloid-Alexa488 and incubated for 20 min at room temperature. Cells were observed and photographed using a fluorescence microscope.

### Cell viability and death analysis

To determine differences in cell viability and death between parental and CBDCA-resistant A2780 cells, three independent assays were performed: (1) cell viability by MTT; (2) phosphatidylserine translocation and (3) caspase-3/7 activation.

Cell viability was evaluated using the MTT-formazan assay as described above. Briefly, cell lines were seeded at a density of 1 × 10^4^ cells in a 96-well plate. After cell attachment, 6.0 µM of CBDCA (EC_50_ of parental A2780 cells) was added to each well plate to evaluate differences in cell viability between parental and CBDCA-resistant A2780 cells.

Phosphatidylserine translocation in cell membrane was determined using Alexa Fluor 488 Annexin V/Dead Cell Apoptosis Kit (Invitrogen, USA) according to the manufacturer’s instructions. A number of 1 × 10^5^ cells were suspended in 100 µL of binding buffer, then 1 µg/mL of propidium iodide (PI) and 5 µL of annexin V were added. Cells were incubated for 15 min at room temperature.

The caspase-3/7 activation analysis was assessed through the CellEvent Caspase/SYTOX AADvanced kit assay (Life Technologies, USA) according to the manufacturer’s indications. Cells were incubated with 500 nM CellEvent reagent and incubated for 25 min at 37 °C. Then, 1 μM of SYTOX AADvanced reagent was added as a counter-tag and incubated for 5 min at 37 °C.

A total of 1.5 × 10^5^ cells were seeded in a 6-well plate. After 24 h, 6.0 µM of CBDCA was added to culture medium. As positive control, cells were treated with 5% dimethyl sulfoxide (DMSO) for 24 h. Cells without treatment were used as a negative control. The measures were analyzed by flow cytometer (FACs Canto II, Becton–Dickinson) using the emission and excitation wavelength suggested by the manufacturer.

### RNA extraction and library preparation

Total cell RNA was isolated from cells using the TRIzol reagent (Life Technologies) according to the manufacturer’s instructions. RNA integrity was checked by Agilent Bioanalyzer 2100. Libraries for RNA-seq were prepared according to KAPA Stranded RNA-Seq Kit with RiboErase (KAPA Biosystems, Wilmington, MA) system. Final library quality and quantity were analyzed by Agilent Bioanalyzer 2100 and Life Technologies Qubit3.0 Fluorometer, respectively. 150 bp Paired-end sequencing was performed on Illumina HiSeq 4000 (Illumnia Inc., San Diego, CA).

### RNA-seq analysis

We analyzed RNA-seq data from the parental A2780 and CBDCA-R-A2780 cell lines using a series of bioinformatics tools and Python (version 3.6.5), R (version 3.3.2), and Bash (version 4.1.2) scripts [69–71].

We first trimmed the RNA-seq reads with Trimmomatic tool (version 0.38) to remove adapter sequences and low-quality reads [72]. Then, we aligned the reads to a reference genome using the sequence alignment tool Kallisto (version 0.44.0) [73]. The human reference genome we used was Release 28 from The ENCODE Project [74]. After aligning the reads, we performed differential expression analysis between the A2780-parental group and the A2780-R-CBDCA group with a program called Sleuth (version 0.30.0) [75]. The scripts we used for this analysis can be found at https://github.com/parkerac/RNA-Seq.

### Function annotation and pathway enrichment of differentially expressed genes

We used PANTHER (http://www.pantherdb.org/) to perform gene ontology (GO) and pathways analysis, using a text file containing a gene ID list and common gene names.

### Statistical analysis

Cell viability and cell death assay data were analyzed by Mann–Whitney test. The EC_50_ values were calculated from dose–response curves; data were analyzed by two-tailed ANOVA with a Bonferroni post-test using GraphPad Prism 5 software (GraphPad, USA). All assays were performed using technical and biological triplicate. A P < 0.05 value was considered statistically significant.

## Additional file

**Additional file 1: Table S1.** Gene list of 156 transcripts.

## Abbreviations

A2780cis: cisplatin-resistant A2780 cell line
CBDCA: carboplatin
EOC: epithelial ovarian cancer
EC_50_: effective concentration that causes 50% cell death
PS: phosphatidylserine
DEGs: differentially expressed genes
GO: gene ontology
sFRP4: secreted frizzled-related protein 4 (sFRP4
COL11A1: collagen 11 alpha 1

## References

[1] Chornokur G, Amankwah EK, Schildkraut JM, Phelan CM (2013). Global ovarian cancer health disparities. Gynecol Oncol..

[2] Gloss BS, Samimi G (2014). Epigenetic biomarkers in epithelial ovarian cancer. Cancer Lett..

[3] Salzberg M, Thurlimann B, Bonnefois H, Fink D, Rochlitz C, von Moos R (2005). Current concepts of treatment strategies in advanced or recurrent ovarian cancer. Oncology..

[4] Alberts DS (1995). Carboplatin versus cisplatin in ovarian cancer. Semin Oncol.

[5] Sandercock J, Parmar MK, Torri V, Qian W (2002). First-line treatment for advanced ovarian cancer: paclitaxel, platinum and the evidence. Br J Cancer.

[6] Chile MDS. Guía Clínica AUGE Cáncer de Ovario Epitelial. Ser Guías Clínicas MINSAL. 1st ed. 2013. p. 26.

[7] Kim A, Ueda Y, Naka T, Enomoto T (2012). Therapeutic strategies in epithelial ovarian cancer. J Exp Clin Cancer Res..

[8] Yan XD, Li M, Yuan Y, Mao N, Pan LY (2007). Biological comparison of ovarian cancer resistant cell lines to cisplatin and Taxol by two different administrations. Oncol Rep.

[9] Niero EL, Rocha-Sales B, Lauand C, Cortez BA, De Souza MM, Rezende-Teixeira P (2014). The multiple facets of drug resistance: one history, different approaches. J Exp Clin Cancer Res..

[10] Kelland L (2007). The resurgence of platinum-based cancer chemotherapy. Nat Rev Cancer.

[11] McDermott M, Eustace AJ, Busschots S, Breen L, Crown J, Clynes M (2014). In vitro development of chemotherapy and targeted therapy drug-resistant cancer cell lines: a practical guide with case studies. Front Oncol..

[12] Van Zyl B, Tang D, Bowden NA (2018). Biomarkers of platinum resistance in ovarian cancer: what can we use to improve treatment. Endocr Relat Cancer..

[13] Fraser M, Leung B, Jahani-Asl A, Yan X, Thompson WE, Tsang BK (2003). Chemoresistance in human ovarian cancer: the role of apoptotic regulators. Reprod Biol Endocrinol..

[14] Shahzad MMK, Lopez-Berestein G, Sood AK (2009). Novel strategies for reversing platinum resistance. Drug Resist Update.

[15] Armstrong SR, Narendrula R, Guo B, Parissenti AM, McCallum KL, Cull S (2012). Distinct genetic alterations occur in ovarian tumor cells selected for combined resistance to carboplatin and docetaxel. J Ovarian Res..

[16] Yan XD, Pan LY, Yuan Y, Lang JH, Mao N (2007). Identification of platinum-resistance associated proteins through proteomic analysis of human ovarian cancer cells and their platinum-resistant sublines. J Proteome Res.

[17] Los G, Verdegaal E, Noteborn HPJM, Ruevekamp M, de Graeff A, Meesters EW (1991). Cellular pharmacokinetics of carboplatin and cisplatin in relation to their cytotoxic action. Biochem Pharmacol..

[18] Beretta GL, Benedetti V, Cossa G, Assaraf YGA, Bram E, Gatti L (2010). Increased levels and defective glycosylation of MRPs in ovarian carcinoma cells resistant to oxaliplatin. Biochem Pharmacol.

[19] Nikounezhad N, Nakhjavani M, Shirazi FH (2016). Generation of Cisplatin-resistant ovarian cancer cell lines. Iran J Pharm Sci..

[20] Colmegna B, Uboldi S, Frapolli R, Licandro SA, Panini N, Galmarini CM (2015). Increased sensitivity to platinum drugs of cancer cells with acquired resistance to trabectedin. Br J Cancer.

[21] Shen D-W, Pouliot LM, Hall MD, Gottesman MM (2012). Cisplatin resistance: a cellular self-defense mechanism resulting from multiple epigenetic and genetic changes. Pharmacol Rev.

[22] Kartalou M, Essigmann JM (2001). Mechanisms of resistance to cisplatin. Mutat Res - Fundam Mol Mech Mutagen..

[23] Galluzzi L, Senovilla L, Vitale I, Michels J, Martins I, Kepp O (2012). Molecular mechanisms of cisplatin resistance. Oncogene..

[24] Dilruba S, Kalayda GV (2016). Platinum-based drugs: past, present and future. Cancer Chemother Pharmacol..

[25] Chen S, Guttridge DC, You Z, Zhang Z, Fribley A, Mayo MW (2001). Wnt-1 signaling inhibits apoptosis by activating β-catenin/T cell factor-mediated transcription. J Cell Biol.

[26] Pećina-Šlaus N (2010). Wnt signal transduction pathway and apoptosis: a review. Cancer Cell Int..

[27] Zhan T, Rindtorff N, Boutros M (2017). Wnt signaling in cancer. Oncogene..

[28] Liu J, Lu J, McKeage M (2012). Membrane transporters as determinants of the pharmacology of platinum anticancer drugs. Curr Cancer Drug Targets..

[29] Prevarskaya N, Skryma R, Shuba Y (2010). Ion channels and the hallmarks of cancer. Trends Mol Med..

[30] Prevarskaya N, Skryma R, Shuba Y (2018). Ion channels in cancer: are cancer hallmarks oncochannelopathies?. Physiol Rev.

[31] Housman G, Byler S, Heerboth S, Lapinska K, Longacre M, Snyder N (2014). Drug resistance in cancer: an overview. Cancers (Basel)..

[32] Keogh JP (2012). Membrane transporters in drug development. Adv Pharmacol.

[33] Litman T, Druley TE, Stein WD, Bates SE (2001). From MDR to MXR: new understanding of multidrug resistance systems, their properties and clinical significance. Cell Mol Life Sci..

[34] Hoffmann EK, Lambert IH (2014). Ion channels and transporters in the development of drug resistance in cancer cells. Philos Trans R Soc Lond B Biol Sci.

[35] Konkimalla VB, Kaina B, Efferth T (2008). Role of transporter genes in cisplatin resistance. Vivo (Brooklyn)..

[36] Kalayda GV, Wagner CH, Buß I, Reedijk J, Jaehde U (2008). Altered localisation of the copper efflux transporters ATP7A and ATP7B associated with cisplatin resistance in human ovarian carcinoma cells. BMC Cancer..

[37] Dmitriev OY (2011). Mechanism of tumor resistance to cisplatin mediated by the copper transporter ATP7B. Biochem Cell Biol..

[38] Eckstein N (2011). Platinum resistance in breast and ovarian cancer cell lines. J Exp Clin Cancer Res..

[39] Nagaraj AB, Joseph P, Kovalenko O, Singh S, Armstrong A, Redline R (2015). Critical role of Wnt/β-catenin signaling in driving epithelial ovarian cancer platinum resistance. Oncotarget..

[40] Pavelka M, Lucas MFA, Russo N (2007). On the hydrolysis mechanism of the second-generation anticancer drug carboplatin. Chem A Eur J..

[41] Rabik CA, Dolan ME (2007). Molecular mechanisms of resistance and toxicity associated with platinating agents. Cancer Treat Rev..

[42] Siddik ZH (2003). Cisplatin: mode of cytotoxic action and molecular basis of resistance. Oncogene..

[43] Rudin CM, Yang Z, Schumaker LM, VanderWeele DJ, Newkirk K, Egorin MJ (2003). Inhibition of glutathione synthesis reverses Bcl-2-mediated cisplatin resistance. Cancer Res.

[44] Arnér ESJ, Nakamura H, Sasada T, Yodoi J, Holmgren A, Spyrou G (2001). Analysis of the inhibition of mammalian thioredoxin, thioredoxin reductase, and glutaredoxin by cis-diamminedichloroplatinum (II) and its major metabolite, the glutathione-platinum complex. Free Radic Biol Med..

[45] Chikazawa N, Tanaka H, Tasaka T, Nakamura M, Tanaka M, Onishi H (2010). Inhibition of Wnt signaling pathway decreases chemotherapy-resistant side-population colon cancer cells. Anticancer Res.

[46] Zhang Z, Cheng L, Li J, Farah E, Atallah NM, Pascuzzi PE (2018). Inhibition of the Wnt/b-catenin pathway overcomes resistance to enzalutamide in castration-resistant prostate cancer. Cancer Res.

[47] He L, Zhu H, Zhou S, Wu T, Wu H, Yang H (2018). Wnt pathway is involved in 5-FU drug resistance of colorectal cancer cells. Exp Mol Med..

[48] Bordonaro M, Tewari S, Cicco CE, Atamna W, Lazarova DL (2011). A switch from canonical to noncanonical wnt signaling mediates drug resistance in colon cancer cells. PLoS ONE..

[49] Tabayashi T, Takahashi Y, Kimura Y, Tomikawa T, Sagawa M, Nemoto T (2014). Targeting the Wnt/beta-catenin signaling pathway in multiple myeloma: a possible new therapeutic approach to overcome bortezomib-resistance. Blood..

[50] Cui J, Jiang W, Wang S, Wang L, Xie K (2012). Role of Wnt/β-catenin signaling in drug resistance of pancreatic cancer. Curr Pharm Des..

[51] Anastas JN, Moon RT (2012). WNT signalling pathways as therapeutic targets in cancer. Nat Rev Cancer.

[52] Zhang W, Liu Y, Sun N, Wang D, Boyd-Kirkup J, Dou X (2013). Integrating genomic, epigenomic, and transcriptomic features reveals modular signatures underlying poor prognosis in ovarian cancer. Cell Rep..

[53] Saran U, Arfuso F, Zeps N, Dharmarajan A (2012). Secreted frizzled-related protein 4 expression is positively associated with responsiveness to Cisplatin of ovarian cancer cell lines in vitro and with lower tumour grade in mucinous ovarian cancers. BMC Cell Biol..

[54] Deshmukh A, Kumar S, Arfuso F, Newsholme P, Dharmarajan A (2017). Secreted Frizzled-related protein 4 (sFRP4) chemo-sensitizes cancer stem cells derived from human breast, prostate, and ovary tumor cell lines. Sci Rep..

[55] Li RN, Liu B, Li XM, Hou LS, Mu XL, Wang H (2017). DACT1 Overexpression in type I ovarian cancer inhibits malignant expansion and cis-platinum resistance by modulating canonical Wnt signalling and autophagy. Sci Rep..

[56] Pfankuchen DB, Baltes F, Batool T, Li J-P, Schlesinger M, Bendas G (2017). Heparin antagonizes cisplatin resistance of A2780 ovarian cancer cells by affecting the Wnt signaling pathway. Oncotarget..

[57] Barghout SH, Zepeda N, Xu Z, Steed H, Lee CH, Fu Y (2015). Elevated β-catenin activity contributes to carboplatin resistance in A2780cp ovarian cancer cells. Biochem Biophys Res Commun.

[58] Imamura Y, Scott IC, Greenspan DS (2000). The pro-alpha3(V) collagen chain. Complete primary structure, expression domains in adult and developing tissues, and comparison to the structures and expression domains of the other types V and XI procollagen chains. J Biol Chem..

[59] Raglow Z, Thomas SM (2015). Tumor matrix protein collagen XIα1 in cancer. Cancer Lett..

[60] Wu YH, Chang TH, Huang YF, Huang HD, Chou CY (2014). COL11A1 promotes tumor progression and predicts poor clinical outcome in ovarian cancer. Oncogene..

[61] Cheon DJ, Tong Y, Sim MS, Dering J, Berel D, Cui X (2014). A collagen-remodeling gene signature regulated by TGF-β signaling is associated with metastasis and poor survival in serous ovarian cancer. Clin Cancer Res..

[62] Li J, Wood WH, Becker KG, Weeraratna AT, Morin PJ (2007). Gene expression response to cisplatin treatment in drug-sensitive and drug-resistant ovarian cancer cells. Oncogene..

[63] Nabavi S (2016). Identifying candidate drivers of drug response in heterogeneous cancer by mining high throughput genomics data. BMC Genomics..

[64] Teng PN, Wang G, Hood BL, Conrads KA, Hamilton CA, Maxwell GL (2014). Identification of candidate circulating cisplatin-resistant biomarkers from epithelial ovarian carcinoma cell secretomes. Br J Cancer..

[65] Wu Y-H, Chang T-H, Huang Y-F, Chen C-C, Chou C-Y (2015). COL11A1 confers chemoresistance on ovarian cancer cells through the activation of Akt/c/EBPβ pathway and PDK1 stabilization. Oncotarget..

[66] Wu YH, Huang YF, Chang TH, Chou CY (2017). Activation of TWIST1 by COL11A1 promotes chemoresistance and inhibits apoptosis in ovarian cancer cells by modulating NF-κB-mediated IKKβ expression. Int J Cancer.

[67] Takahashi-Yanaga F, Kahn M (2010). Targeting Wnt signaling: can we safely eradicate cancer stem cells?. Clin. Cancer Res..

[68] Fischer MM, Cancilla B, Yeung VP, Cattaruzza F, Chartier C, Murriel CL (2017). WNT antagonists exhibit unique combinatorial antitumor activity with taxanes by potentiating mitotic cell death. Sci Adv..

[69] Python Software Foundation. Python Language Reference, version 3.6.5. 2016.

[70] Team RDC, R Development Core Team R. R: a language and environment for statistical computing. R Found Stat Comput. 2016.

[71] Free software Foundation. Bash (4.2.1). 2009. http://gnu.org/licenses/gpl.html.

[72] Bolger AM, Lohse M, Usadel B (2014). Trimmomatic: a flexible trimmer for Illumina sequence data. Bioinformatics.

[73] Bray NL, Pimentel H, Melsted P, Pachter L (2016). Near-optimal probabilistic RNA-seq quantification. Nat Biotechnol..

[74] Harrow J, Frankish A, Gonzalez JM, Tapanari E, Diekhans M, Kokocinski F (2012). GENCODE: the reference human genome annotation for the ENCODE project. Genome Res..

[75] Pimentel H, Bray NL, Puente S, Melsted P, Pachter L (2017). Differential analysis of RNA-seq incorporating quantification uncertainty. Nat Methods..

